# Nationwide consensus on quality indicators to assess glaucoma care: A modified Delphi approach

**DOI:** 10.1177/11206721231170033

**Published:** 2023-04-17

**Authors:** Flavio Iorio-Aranha, Cláudia de Freitas, Amândio Rocha-Sousa, Ana Azevedo, João Barbosa-Breda

**Affiliations:** 1 EPIUnit, Institute of Public Health, University of Porto, Porto, Portugal; 2 Laboratório para a Investigação Integrativa e Translacional em Saúde Populacional (ITR), Porto, Portugal; 3 Ophthalmology, Faculty of Medicine, Universidade de Brasilia, Brasilia, Brasil; 4 Department of Public Health and Forensic Sciences and Medical Education, Faculdade de Medicina da Universidade do Porto, Porto, Portugal; 5 UnIC@RISE, Department of Surgery and Physiology, Faculdade de Medicina da Universidade do Porto, Porto, Portugal; 6 Department of Ophthalmology, Centro Hospitalar Universitário São João, Porto, Portugal; 7 Hospital Epidemiology Center, Centro Hospitalar Universitário São João, Porto, Portugal; 8 Department of Neurosciences, KULeuven, Research Group Ophthalmology, Leuven, Belgium

**Keywords:** Glaucoma, quality indicators, quality of care, consensus methods, Delphi technique

## Abstract

**Purpose:**

Performance assessments are essential to tracking and improving quality in health care systems. Key aspects of the care process that act as indicators must be measured in order to gain an in-depth understanding of a care unit's operation. Without standardized quality indicators (QIs), it is difficult to characterize and compare the abilities of institutions to achieve excellence. The aim of this study is to reach a consensus among glaucoma specialists concerning the development of a set of QIs to assess the performance of glaucoma care units.

**Methods:**

A two-round Delphi technique was performed among glaucoma specialists in Portugal, using a 7-point Likert scale. Fifty-three initial statements (comprising process, structure, and outcome indicators) were evaluated and participants had to agree on which ones would be part of the final set of QIs.

**Results:**

By the end of both rounds, 28 glaucoma specialists reached consensus on 30/53 (57%) statements, including 19 (63%) process indicators (mainly relating to the proper implementation of complementary exams and the setting of follow-up intervals), 6 (20%) structure indicators, and 5 (17%) outcome indicators. Of the indicators that were part of the final list, functional and structural aspects of glaucoma progression and the availability of surgical/laser procedures were the most prevalent.

**Conclusions:**

A set of 30 QIs for measuring the performance of glaucoma units was developed using a consensus methodology involving experts in the field. Their use as measurement standards would provide important information about unit operations and allow further implementation of quality improvements.

## Introduction

In order to understand and evaluate the overall performance of a health care system, it is necessary to analyze its operational processes and outcomes over time. In addition to providing for a better quality assessment and improved accountability, this analysis would allow for comparisons between different health care systems. Such an assessment requires a monitoring system supported by appropriate indicators, that enable the classification and comparison of performance results.^1^

Quality Indicators (QIs) are derived from measurable aspects of clinical practice that reflect a system's operation and can be used to track clinical performance. QIs are commonly classified in 3 categories: structure (regarding material and human resources), process (the way health care is delivered to patients), and outcome (patient health status as a result of care received).^2^ QIs represent specific attributes of care and allow for continuous re-assessment after changes are implemented, thus contributing to quality improvement.^3^ It is also important that the measurable aspects of analyzed QIs are valid, reliable, and universally accepted. Their development should combine the best scientific evidence available with insights from clinical practice, and consensus methodologies are one of the most often used techniques to accomplish this task.^4^

In the health care science field, many aspects regarding measurement methods have not yet reached consensus or a sufficient body of evidence to be standardized. Some efforts have been made to address this issue using instruments to reach consensus between stakeholders,^5^ like COMET (Core Outcome Measures in Effectiveness Trials) and COSMIN (Consensus-based Standards for the Selection of Health Measurement Instruments).^6,7^

In ophthalmology, QIs have been developed in areas like cataract^8^ and macular degeneration.^9^ However, in the field of glaucoma, standardized outcomes are mostly limited to clinical trials.^10,11^

Glaucoma is the leading cause of irreversible blindness in the world.^12^ It is a chronic disease, with almost no signs or symptoms, and appropriate treatment can slow its progression. The number of people affected by glaucoma is increasing as the general worldwide population ages, straining the health system with the growing demand for glaucoma care. There is a current trend in glaucoma care of seeking new and highly efficient health management models that can deal with such high demand.^13^ In this context, well-designed QIs are important instruments for comparing health units, gauging different models of care, and overseeing the impact of any changes that have been implemented.

In addition to monitoring the final results of health care via the use of outcome indicators, a comprehensive evaluation of how care is provided is essential. For this reason, aspects of structure and process indicators must be included in the analysis. This study intends to develop a set of QIs through a nationwide consensus panel that can be used to evaluate health care performance in glaucoma units. To that end, a modified Delphi approach will be adopted involving a group of Portuguese ophthalmologists who are especially dedicated to glaucoma.

## Methods

A two-round modified Delphi technique was used in this study. All steps of the process were stated *a priori* and the methodology followed recommendations described in the literature.^14,15,16^ The Delphi technique is a method for reaching consensus on a controversial or poorly studied topic. It is often used when there is a lack of evidence-based information and one has to rely on the opinion and experience of specialists to support decisions. It consists in a few rounds of questionnaires with individual feedback that provide a unique way of interaction among the panel members, avoiding direct contact and preserving anonymity.^16^

In order to develop the first list of potential QIs, a broad literature review was performed by the authors (July 2020), using the expression “glaucoma AND quality indicators AND performance” in the following databases: MEDLINE(Pubmed), Cochrane and Web of Science. From the initial 1177 records identified, 53 studies were selected for full evaluation. The authors intended to search for indicators that were used in performance assessment of glaucoma care units. Most of the studies assessed focused on core outcome measures used in clinical trials.^10,15,17^ Specific studies about performance QIs were scarce^18^ (n  =  9 studies). Nevertheless, the team members’ knowledge acquired during the development of a recent scoping review^18^ about the specific subject of this study turned out to be a good basis for the development of QIs in glaucoma care. The literature review was supplemented by an assessment of the latest updated international guidelines for glaucoma at the time of the review (from the American Academy of Ophthalmology-AAO (published in 2015), the European Glaucoma Society-EGS (4th edition - 2014), the National Institute for Health and Care Excellence-NICE (published in 2017), the Canadian Ophthalmological Society (published in 2009) and the Finnish Current Care Guideline for Glaucoma (published in 2014)). Information about the main domains to be captured by performance QIs in glaucoma was then retrieved from all the studies and guidelines selected and analyzed (a detailed list of the process domains identified is available in the scoping review^18^). Finally, the authors (steering group) proposed one or more specific indicators for each domain.

A group of 45 ophthalmologists recognized for being glaucoma experts (current members of the Portuguese Glaucoma Group, representing the whole country) were invited to participate in the study by e-mail, through the Portuguese Glaucoma Group contact list. Participation was strictly voluntary and anonymous for all participants during both rounds. Consent for participation was requested and obtained from all participants: access to the online platform displaying the QIs’ statements was subject to participants’ actively ticking consent for participation.

Each of the two Delphi rounds took 3 weeks to complete, with a 3-week break between them. The first round took place in February 2021. The questionnaires were created and distributed using Google Forms (Google LLC, USA), a web-based survey tool. They consisted of a list of statements, with each statement corresponding to a possible glaucoma QI. All communication with participants was done by e-mail, including two reminders sent 1 week after each questionnaire was originally sent.

The participants (panelists) were asked to rate the statements according to their level of agreement with the inclusion of each corresponding QI statement in the set of indicators used to assess the performance of glaucoma units. The process was based on a 7-point Likert scale (ranging from 1-strongly disagree to 7-strongly agree). They were also asked to contribute with comments and suggestions about new indicators that they considered important and which could be added to the second round. As an example, in case of the possible QI “Does the glaucoma department have surgical procedures available?” presented to participants’ evaluation, the intention of this QI/question is to know if the information provided by that QI (e.g., the department having or not surgical procedures) is important enough to be used as an indicator of the level of performance of a glaucoma unit. This is irrespective of the answer being “yes” or “no” when such QI is applied in real practice (once a final set is decided). For this paper, the participants/experts were asked to express their opinion about the inclusion of the QI, declaring how much they agree or disagree with the importance of that information being used as a QI the final set.

In the first round, 50 statements were sent to participants and information on participants’ characteristics (age and years of experience in glaucoma care) was also collected. The statements were grouped in the 3 categories proposed by Donabedian (1988)^2^: structure indicators with 9 statements, process indicators with 26, and outcome indicators with 15. The survey was preceded by an explanation about the scope of the study, what is meant by QIs and how they are used to assess performance within a health care service, in order to level group understanding. Instructions on how to use the Likert scale were also included.

After the first round, the questionnaire's responses were evaluated by the steering group. Descriptive analyses were performed in order to assess the panelists’ opinion (median and 25th/75th quartiles). New suggestions that came from participants were also analyzed by the steering group in order to assess their eligibility to become new statements in the second round. Consensus in all rounds was defined by agreement of the participants around a specific statement, through a method of proportion within a restricted range.^15^ Since this study anticipated a certain degree of homogeneity in the participants’ responses,^10^ a more restrict range was chosen for achieving consensus rather than the usual cut-off limit (scores **≥** 5 on the 7-point Likert scale).^19^ Consensus was reached when 75% or more of the participants scored the statement 6 or 7 in the Likert scale.^20^ Then, percentage of participants that scored **≥**5 and **≥** 6 in the scale were calculated and presented.

The second round was comprised of the statements that did not reach consensus in the first round, plus the additional proposed statements. Feedback regarding the results from the first round was also provided, including a comparison between each participant's answers and the average of the whole group. In order to maintain anonymity from other participants, the surveys were individualized at this stage. Any changes on answers to the statements that had not reached consensus were permitted during the second round.

After both rounds, a new statistical analysis was done and a list of QIs that reached consensus was compiled. In order to assess the reliability of panelists’ responses, the Cronbach's alpha coefficient, which estimates the reliability of a psychometric instrument, through the measurement of its internal consistency, was calculated.^21^

## Results

Among the 45 glaucoma experts invited, 32 (71%) responded to the first round. In the second round, 28 (62%) experts participated, corresponding to a drop-out rate between rounds of roughly 12%. Characteristics of participants are described in Table 1. The majority of participants (84%) had more than 6 years of experience in glaucoma and 62% were younger than 50 years old.

**Table 1.** Characteristics of participants in the Delphi panel to reach consensus about glaucoma quality indicators.

	N (%)
Participants invited	45 (100%)
Participants in 1st round	32 (71%)
Participants in 2nd round	28 (62%)
Age of participants	
30-39	10 (31%)
40-49	10 (31%)
50-59	6 (19%)
≥ 60	6 (19%)
Years of experience in glaucoma	
< 5	5 (16%)
6-10	7 (22%)
11–20	10 (31%)
≥ 20	10 (31%)

Of the 50 initial statements presented to participants, 23 reached consensus in round 1, meaning that 75% or more of the panelists scored them with 6 or 7 points (Table 2).

**Table 2.** Glaucoma quality indicators (results of a Delphi procedure using a 7-point Likert scale^1^).

Indicators	1st round^2,3^	2nd round^2,4^	Final consensus^5^	% Panelists scoring^6^	
				≥5	≥6
**Structure**					
Does the glaucoma department/unit have Nd:YAG laser therapy available?	7 (7–7)		☑	97%	91%
Does the glaucoma department/unit have surgical procedures available?	7 (7-7)		☑	97%	94%
Number and types of equipment in glaucoma department/unit	7 (6-7)		☑	91%	88%
Glaucoma patients per doctor in glaucoma department/unit	7 (6-7)		☑	91%	84%
Does the glaucoma department/unit have a laser with SLT (Selective Laser Trabeculoplasty) capability available?		6 (6–7)	☑	96%	79%
Does the glaucoma department/unit have Argon/Argon-like laser therapy available?	7 (5–7)	6 (6–7)	☑	89%	75%
Ratio of orthoptists by the number of ophthalmologists in the glaucoma department/unit.		6 (5–7)		79%	61%
Does the glaucoma department/unit have an Electronic Health Record (EHR) implemented?	6 (5–7)	6 (5–6)		89%	57%
Does the glaucoma department/unit have a comprehensive written protocol for all aspects of glaucoma care (interval times, thresholds for abnormal parameters, workflow and sequence of procedures, etc.)?	6 (5–6)	6 (5–6)		89%	57%
Number of ophthalmologists in glaucoma department/unit	6 (5–6)	6 (5–6)		82%	57%
Does the glaucoma department/unit have social assistance available to patients?	5 (4–6)	5 (5–6)		79%	39%
**Process**					
Percentage of patients that had the Gonioscopy performed at the time of first diagnosis	7 (7–7)		☑	97%	94%
Percentage of patients whose disease progression was regularly assessed according to the time interval proposed by the guideline/protocol used by the glaucoma department/unit	7 (7–7)		☑	97%	94%
Percentage of patients who have their IOP checked according to the time interval proposed by the guideline/protocol used by the glaucoma department/unit	7 (7–7)		☑	97%	91%
Percentage of patients that had the Visual Field Test performed according to the time interval proposed by the guideline/protocol used by the glaucoma department/unit	7 (7–7)		☑	97%	91%
Waiting time for the first appointment with the Glaucoma Specialist at the glaucoma department/unit	7 (6–7)		☑	91%	91%
Percentage of patients that had the Follow-up Interval performed according to the guideline/protocol used by the glaucoma department/unit	7 (6–7)		☑	97%	91%
Percentage of patients that had Risk Factors for glaucoma documented	6,5 (6–7)		☑	94%	91%
Percentage of patients that had the Fundus examination done according to the time interval proposed by the guideline/protocol used by the glaucoma department/unit	7 (7–7)		☑	97%	88%
Percentage of patients that had the Stage of Disease Severity documented	7 (6–7)		☑	97%	88%
Percentage of patients that had the Retinal Nerve Fiber Layer Structural exam (OCT or similar technology) done according to the time interval proposed by the guideline/protocol used by the glaucoma department/unit	7 (6–7)		☑	94%	84%
Percentage of patients that had the Target IOP documented	6 (6–7)		☑	94%	84%
Percentage of patients that had the Slit-lamp biomicroscopy exam performed according to the time interval proposed by the guideline/protocol used by the glaucoma department/unit	7 (6–7)		☑	91%	84%
Number of patients treated in glaucoma department/unit	6 (6–7)		☑	91%	84%
Percentage of patients that had the Gonioscopy performed according to the time interval proposed by the guideline/protocol used by the glaucoma department/unit	7 (6–7)		☑	94%	81%
Percentage of patients that had their Target IOP redefined according to the time interval proposed by the guideline/protocol used by the glaucoma department/unit	6 (6–7)		☑	94%	81%
Percentage of patients that had the Optic Nerve Head Morphology evaluation (retinography, OCT or similar technology; not fundoscopy) done according to the time interval proposed by the guideline/protocol used by the glaucoma department/unit	7 (6–7)		☑	91%	81%
Percentage of patients that had the Central Corneal Thickness measured at the time of first diagnosis	7 (6–7)		☑	91%	81%
Percentage of patients that had the Visual Acuity measured according to the time interval proposed by the guideline/protocol used by the glaucoma department/unit	6 (6–7)		☑	91%	78%
Percentage of patients who have been the subject of strategies to improve adherence to treatment	6 (6–7)	6 (6–6)	☑	96%	75%
Waiting time for the first appointment with a General Ophthalmologist at the Ophthalmology Department	6 (5–7)	6 (5–6)		75%	68%
Percentage of patients who have been the subject of strategies to improve their glaucoma related literacy	6 (5–7)	6 (5–6)		89%	64%
Percentage of patients that had “vision related quality of life” or “quality of life” parameters measured	5 (5–6)	5 (5–6)		89%	43%
Does the glaucoma department/unit have a way to evaluate team indicators (inter-professional teamwork performance, job satisfaction, etc.)?	5 (4–6)	5 (5–6)		79%	43%
Does the glaucoma department/unit have a way to evaluate aspects of patient safety (harm caused to patient by errors or adverse events)?	5 (5–6)	5 (5–6)		89%	39%
Percentage of patients that had the Assessment for Relative Afferent Pupillary Defect performed according to the time interval proposed by the guideline/protocol used by the glaucoma department/unit	5 (5–6)	5 (4–6)		71%	32%
Percentage of patients that performed a diurnal variation IOP curve	5 (3–6)	5 (3–5)		57%	21%
**Outcome**					
Percentage of patients that have achieved successful results in glaucoma surgery (success defined according to the protocol used by the glaucoma department/unit)	7 (6–7)		☑	97%	91%
Percentage of patients that achieved a stable function/Visual Field (no progression) (including clinical judgment; trend analysis - MD, VFI; pointwise analysis; or specifically developed score)	6 (6–7)	6 (6–7)	☑	93%	79%
Percentage of patients that have achieved a stable structure (no progression) (including ONH, macular or RNFL parameters; through fundus examination, photographs, OCT, or HRT)	6 (5–7)	6 (6–6)	☑	93%	75%
Percentage of patients that have complied with the use of IOP-lowering ocular medication	6 (5–6)	6 (6–6)	☑	93%	75%
Percentage of patients with surgical complications		6 (6–6)	☑	93%	75%
Percentage of patients that have achieved Target IOP (target IOP defined according to the protocol used by the glaucoma department/unit or national/international guidelines)	6 (5–7)	6 (5–6)		93%	71%
Percentage of patients that have achieved X% reduction of IOP from baseline using GAT (X% defined according to the protocol used by the glaucoma department/unit or national/international guidelines)	6 (5–6)	6 (5–6)		89%	68%
Percentage of patients that have achieved successful results in laser trabeculoplasty (success defined according to the protocol used by the glaucoma department/unit)	6 (5–7)	6 (5–6)		86%	61%
Percentage of patients that have improved their scores in “vision related quality of life” or “quality of life” parameters/questionnaires	6 (5–7)	6 (5–6)		89%	61%
Percentage of patients that have become bilaterally totally blind (VA no light perception)	5,5 (5–6)	5,5 (5–6)		82%	50%
Percentage of patients with worsening best-corrected visual acuity considered to be only due to glaucoma (not cataract, macular degeneration or other cause)	6 (5–6)	5,5 (5–6)		82%	50%
Percentage of patients that have become bilaterally legally blind (VA <1/10 or visual field less than 10°)	5,5 (5–6)	5 (5–6)		82%	46%
Percentage of patients in whom a disc rim hemorrhage was found during the course of disease	5 (3–6)	5 (4–6)		68%	36%
Percentage of patients with narrower angle comparing with previous gonioscopic exams	4 (3–5)	4 (3–5)		43%	14%
Percentage of patients that had an increase in IOP-lowering ocular medication	4,5 (3–6)	4,5 (3–5)		50%	14%
Percentage of patients with darker Trabecular Pigmentation comparing with previous gonioscopic exams	4 (3–5)	4 (3–5)		39%	7%

1. Likert scale categories: 1-“Strongly disagree”; 2-“Moderately disagree”; 3-“Slightly disagree”; 4-“Indifferent”; 5-“Slightly agree”; 6-“Moderately agree”; 7-“Strongly agree”.

2. Results from 1st and 2nd rounds are described through median and interquartile range, as “median (Q1-Q3)”; Q1 – first quartile (25% of the answers are below this point); Q3 – third quartile (75% of the answers are below this point); Quartiles were rounded to the nearest whole number. For instance, a result of “6(5–7)” means that the statement reached a median agreement of 6 among the possible answers of the 7-point Likert scale - from 1-Strongly disagree and 7-Stongly Agree, i.e., 50% of panelists rated 6 or lower. And the numbers inside the parentheses (5–7) represent that 25% of the participants rated this statement with up to 5, while the top 25% of panelists’ rating was 7 (strongly agree), representing the first and third quartile, respectively*.*

3. Blank spaces in this column means that the indicator was added from a participant suggestion only to the second round*.*

4. Blank spaces in this column means that the indicator reached consensus already in the first round and was not evaluated in the second round.

5. Final consensus was reached when 75% or more of the participants scored 6 or 7 in the Likert scale, in round 1 or 2. The QIs selected following this criteria are marked with ☑.

6. Percentage of participants that chose a specific group of scores in the Likert scale. Group (≥5) for scores 5, 6 or 7. And group (≥6) for scores 6 or 7. Percentages are related to round 2, except for statements that reached consensus in round 1 or those only added to round 2.

Regarding structure QIs, of the 9 initial statements, 4 (44%) reached consensus in round 1. Two new indicators/statements were added in the second round as the result of participants’ suggestions. In round 2, two additional indicators reached consensus.

In the sub-group of process QIs, of the original 26 statements, 18 (69%) reached consensus in round 1. No new indicators were presented in round 2. Only 1 further indicator reached consensus in round 2.

As for outcome QIs, of the 15 initial statements, only 1 (7%) reached consensus in round 1. One new indicator was added in round 2. Four more indicators reached consensus in round 2.

In total, participants assessed 53 indicators, including three indicators proposed by participants themselves. From these, 30 (57%) reached consensus among glaucoma experts by the end of both rounds.

In Table 2, all statements and their individual results regarding each round (median and interquartile range) are presented. In addition, the exact percentage of participants who scored statements with 6 or 7 (≥ 6) in the Likert scale (attaining consensus when reaching ≥ 75% of participants) are also presented. Another information presented is the percentage of participants who scored 5, 6 or 7 (≥ 5) in the Likert scale (reflecting the amount of participants who considered the statement at least “slightly” important).

The Cronbach's alpha coefficient was calculated for both rounds. It was 0.94 in round 1 and 0.92 in round 2.

## Discussion

By the end of both rounds of this study, a nationwide distribution of 28 Portuguese ophthalmologists with expertise in glaucoma participated in the development of a set of QIs that can be used to assess the performance of glaucoma units. These experts reached a consensus on 30 out of the 53 QIs presented for evaluation, including 19 process indicators (73% of the 26 process indicators proposed), 6 structure indicators (67% of the 9 structure indicators proposed) and 5 outcome indicators (33% of the 15 outcome indicators proposed).

With respect to glaucoma, the way care is delivered and the patient's adherence to treatment are of paramount importance for the prognosis of the disease.^22^ Following reference guidelines regarding treatment and follow-up has an impact on patients’ vision-related quality of life.^23^ Some of the main issues identified in glaucoma clinical practice are not usually the result of limitations in the sensitivity or specificity of exams to identify disease progression, nor the result of ineffective medications. Instead, they are often linked to flaws in the glaucoma care process (including poor patient adherence to treatment,^24^ substandard care,^25^ delays in diagnosis and treatment,^26^ PIO fluctuations during treatment,^27^ and administrative errors in following the proper time intervals between appointments^28^). For this reason, the performance assessment of the system and the way care is provided are currently the most important aspects of glaucoma care to be considered in order to improve outcomes.

Measurement standards in the field of glaucoma are lacking, and it is imperative to develop appropriate indicators for conducting a precise evaluation of the health care system's performance.^29^ Only by comparing the results of indicators that measure the same thing, within a single health care unit or across different ones, is it possible to properly identify problems and constraints in the workflow. This is key to enabling the implementation of changes that will improve effectiveness, achieve process optimization, reduce costs, and minimize safety hazards.

Fully operational QIs also make it possible to continuously monitor quality changes, and consequently, to continuously reassess any improvements that have been implemented. This is also important in tracking changes in sickness patterns over time, and for real-time warnings about sudden, unexpected events, such as the current COVID-19 pandemic crisis.

From the various consensus methods used to standardize procedures and recommendations (expert panel meetings, Delphi surveys, Nominal Group Techniques, focus groups, individual interviews, and individual questionnaires), the Delphi Technique is one of the most used due to its feasibility, reliability, validity, and structured features.^14,30^

The lack of face-to-face interaction and the possibility of web-based communication afforded by the Delphi technique not only allow to gather information from people who are geographically distant, it also proved invaluable to overcome access constraints caused by the COVID-19 pandemic. Moreover, it enables participants to remain anonymous to each other reducing potential for influence by more persuasive participants, which is ideal to reach consensus on a poorly explored issue.

In this study, one interesting aspect observed was the discrepancy between how many process indicators reached a consensus in comparison to the other 2 types of QIs. At the last round, from the 30 indicators selected, 19 (63%) were process indicators, 6 (20%) were structure indicators, and 5 (17%) were outcome indicators. This might be explained by the fact that process indicators make it easier to evaluate the system's performance through the observation of how the system is operating at a particular moment, rather than evaluating the final results of care (measured by outcome indicators).^31^ It is indeed more intuitive to think about process indicators when evaluating how the work is proceeding on a daily basis, rather than outcome indicators that are likely to make more sense to consider when assessing results in clinical trials.

Another key finding of this study was that QIs more specifically related to the patient as an individual (quality of life, patient literacy, safety, social assistance, visual impairment, and visual acuity characteristics) did not reach a formal consensus, despite the growing importance of such issues. However, 79% or more of participants considered these QIs at least “slightly” important (≥ 5 in Table 2), which could denote a tendency toward a greater importance currently being given to these types of QIs.

Although only 11% of participants disagreed with the use of electronic health records (EHRs) as an important instrument to monitor quality of care, this statement did not reach formal consensus. EHRs are very useful tools to quickly access information and statistics related to system performance.^32,33^ However, the not so user-friendly design of the softwares currently available in the market is probably being seen as an obstacle to a free and fast workflow, which causes apprehension among users. Moreover, physicians that are still using paper charts might be resistant to switching to EHRs.

Glaucoma best practices already have a good degree of homogeneity of opinions presented in guidelines and, therefore, the use in this study of a stronger criteria for consensus (75% or more participants scoring 6 or 7 in a 7-point Likert scale^19,34,35^) was a major benefit rather than the traditional 75% or more scoring 5, 6 or 7.^36^

An important characteristic of this study was the listing of all statements with their respective scores. This data presentation strategy (Table 2) allows comparison among QIs and the analyses of how much participants really agreed with each other. This can be very useful for further evaluation of which QIs are best suited for different glaucoma units, according to local particularities and necessities.

Another strength of this study was that the 7-point Likert scale used has shown good indices for reliability, validity, discriminating power, respondent preferences and internal consistency.^37^ Furthermore, this study was conducted according to the methodological quality criteria for reporting Delphi studies recommended in a recent systematic review,^15^ resulting in high face and content validity for the indicators.^31^

The low drop-out rate (only 12% of participants between rounds), and the fact that the pool of invited participants represents the majority of glaucoma experts from an entire country (Portugal), also supports good representativeness for the consensus strategy used.

This study showed a low error variance. The Cronbach's alpha was > 0.9 in both rounds, which is considered excellent.^38^

Limitations of this work also exist. It represents the reality of one single country and cannot be directly translated to other health care systems. Opinions from patients and other stakeholders were not evaluated, which can explain the low presence of patient-reported and financial QIs among the final list. Participants were not asked to declare any potential conflicts of interest. Although this is a limitation of the study, it has nevertheless been mitigated by ensuring participants’ anonymity and the strict confidentiality of their statements.

When attempting to compare to other reports in the area of glaucoma care, it becomes clear how scarce they are. Also, the majority of them only concerned glaucoma outcome indicators, whether for use in clinical trials^10,11^ or to evaluate patient-reported outcomes.^39^ Even fewer studies address indicators that represent the following of established guidelines (also called appropriateness of eyecare delivery).^40^ This fact hindered the possibility of making sound comparisons between the results of this study and of previous ones. QIs that assess performance of a glaucoma unit, through structure, process and outcome indicators, are even more difficult to find, and essentially were represented by two institutional quality standards documents (NICE - National Institute for Health and Care Excellence from United Kingdom^41^ and Health Quality Ontario from Canada^42^) and a recent scoping review^18^ that summarized and created a comprehensive list of process QIs domains in glaucoma care. The list of QIs chosen in this current study are in accordance with the list from the cited scoping review and the two quality institutions, denoting the vision towards convergence to uniformity around this subject.

In conclusion, a nationwide panel of glaucoma specialists agreed on 30 QIs that can be used in the performance assessment of glaucoma units. This list covers the main aspects of glaucoma health care regarding structure, process and outcome indicators. Future research should refine and validate these QIs, in order to achieve a broad, ideally international, standardization among glaucoma indicators.
